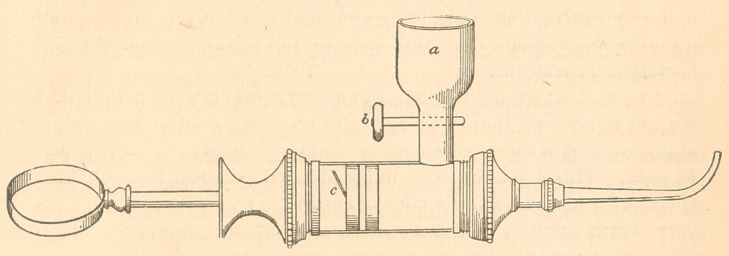# Bleaching of Discolored Dentine Practically Considered

**Published:** 1890-02

**Authors:** E. P. Wright

**Affiliations:** Richmond, Va.


### BLEACHING OF DISCOLORED DENTINE PRACTICALLY CONSIDERED.[1]

BY E. P. WRIGHT, D.D.S., RICHMOND, VA.

In the report of the proceedings of the March meeting of the First District Dental Society of New York there appeared a paper which was most scientifically discussed,—a method of bleaching discolored dentine, by Dr. Kirk, of Philadelphia, in which he gave the credit of inventing the apparatus, there used, to myself.

I do not design nor wish to make an elaborate or scientific defence of this method; that Dr. Kirk has done sufficiently; but I simply desire to set up claims for the restoration of discolored crowns to comparative usefulness by bleaching and filling, as against decapitation and crowning. To ratify these claims it is obviously necessary that certain prejudices, in the minds of a large class of practitioners, should be done away with.

First of all, our experience has proved, beyond question, that dentine can be bleached without subsequent re-discoloration. To this proposition, however, we allow consideration for that state of peculiar discoloration which is so familiar to us all, the color of which is from a light to a very dark yellow. The cause of this discoloration seems to be attributed, by the profession, to some defective or peculiar arrangement of the dentinal tubuli, which takes place during the stages of calcification.

When two years ago I began a series of experiments, with a view to the application of free chlorine and other bleaching agents to discolored dentine, I found such insufficient data to go upon, that my labors were crowned with poor success, and at the same time proving an expensive thing to my patients; as I candidly admit that I ruined several teeth by making them darker than they were before I began treatment. I trust, however, that the victims on the altar of science may be properly resigned, and have no malice stored up for me.

When we are able to make up our minds to the importance of

[1] Read at the opening meeting of the Odontological Society of Pennsylvania, October 5, 1889.

preserving the tooth-structure, even when it is not so sightly as we might desire, and refrain from removing the natural tissue for a questionable substitute, we will have come to a wise conclusion.

With great respect for those who are madly rushing to porcelain teeth, I would say that they are doing the profession an injury, as there is no higher glory for one who professes the healing art than that of preserving the natural tissue. This tendency to porcelain crowns and bridge-work, in its many phases, is going to have, ultimately, the same effect upon the profession and the public that the introduction of a plastic base plate has had.

I would place the porcelain crown on the one hand, and the vulcanite base on the other,—they stand in the same relation to the advancement of our profession. We all regret the universal favor which has been accorded the vulcanite base, and rather look forward, I think, to the time when we shall have a reliable method by which we can retain the natural organs. Are we not taking a step in the right direction by aiming to accomplish this, and thus put a curb on that bent of our disposition to a retrograde movement?

A student of dentistry, looking through our journals, would be inclined, I imagine, to the impression that there was little else in dentistry than crowning and bridge-work. This is wrong; and while it is true that all plans are made clearer and plainer by discussion and illustration, yet too great emphasis on any theory is hurtful to the people.

In advocating the retention of the natural crown, at a sacrifice of symmetry and beauty, compared with an artificial one, we would avoid this error, desiring only to present to you the intrinsic merit of the question, stimulated by a firm conviction that, if we do our duty in preserving the natural organ, it will be a gain to the profession and keep us on the upper plane.

While it is an easy matter to bleach discolored dentine and preserve the natural crown in good shape, still it must be admitted that it is easier to remove the natural crown and substitute a porcelain one; but, as before stated, it is not well, nor is it to the interest of the highest aims of our calling, to consider too much the matter of ease or other minor effects, either to the patient or the operator; and as we are considering this subject from a practical point of view, we should look at it in that way, because, even though the individual efforts may not be so beautiful or, at first, so satisfactory from an æsthetic point, still we have satisfied our conscience, we are teaching our patients the importance of the natural organs, and encouraging an inventive and upward tendency among our brethren. We are maintaining the standard set for us by our great men; in short, we are doing the best we can.

Before discussing the matter in the light of a valuable principle, we want to know what has been accomplished.

In the outset we state our positive belief that teeth properly treated—and certainly those amenable to the chlorine treatment—do not re-discolor; this assertion is based upon experience extending over two years; and, after many experiments, so far, I have not had a single re-discoloration where I have succeeded in bleaching. In my experience, the yellowish condition, of which mention is made at the beginning of the paper, has been very difficult of correction. It may be well to state that the present literature on the subject favors the idea that this latter peculiar discoloration can be removed by the use of dilute sulphuric acid, and there are many methods by which it is applied. My prejudice to sulphuric acid or any other acid known to act upon, in a deleterious sense, the organization of the tooth-structure has prevented me from experimenting, to any degree, in that particular line; therefore I cannot enlighten you upon the uses of sulphuric acid as applied to bleaching teeth. Saying this much of this peculiar discoloration, which is a yellowish mud-color, and of which we know so little in a remedial sense, and of the supposed activity of the agent (sulphuric acid), we pass on to the discussion of that class of discolored dentine with which we are familiar, and of which we know something positive.

Very lately, through the assistance of an eminent chemist, Dr. Froehling, I have succeeded in formulating a theory as to the cause of these peculiar discolorations. The value of this theory is questionable, and I hesitate to advance it. It may be well, however, to state in general terms that these discolorations can only be organic and perhaps of a fatty nature, and our failure to remove it heretofore has probably been due to the inadaptability of the method employed to destroy the fatty matter contained in the tubuli. I regret that this conclusion should not have been arrived at sooner. I have now, however, formulated a plan by which it seems there will be no difficulty in the future in thoroughly removing every particle of fatty matter from the tubes.

That you may understand more clearly the plan I wish now to place before you, I will hand around the apparatus to be used in the first steps towards the treatment of discolored teeth, as aforesaid. The instrument consists simply of an exhaust-pump, made on a small scale, of a simple retort connected with the barrel in which the piston works; the contents of the retort are controlled by a stop-cock which governs the flow of whatever chemical it may contain. The agent recommended to be used is ether, chloroform, or petroleum ether (rhigolene), either one of which will act powerfully upon and dissolve the fatty globules. This apparatus must have its point in serted into a preparation such as is used in the hectograph, a material which is not affected by either of the three agents mentioned; it is, however, easily acted upon by another of our favorites, ammonia, and thus *its* use is debarred.

I believe the hectograph composition can be made by putting pure glue in cold water, leaving it until it swells, draw off the water, melting it in its absorbed water, and mixing about six parts of the glue solution to one of glycerin. This compound is intended to be used after the rubber dam has been placed around the tooth and the tooth made dry, that the rubber dam, which is designed to keep back the moisture, shall not be interfered with by the action of the chemical agent employed.

The *modus operandi*, with the exception of the incompleteness of preparatory treatment, as will be shown later on, has been clearly described by Dr. Kirk, and published in the *Cosmos*. I shall therefore not take up your time in repeating it, but will close my paper with the proposition that ninety-five per cent. of discolored teeth can be bleached in from not more than one to three sittings of one hour each. Of course, preparatory treatment is necessary, and precaution also in regard to the use of oleaginous substances within the canal. If such have been used, from one to two sittings, close upon each other, will be required, and the oily substance previously removed as before indicated.

Of the two or three failures, strictly speaking, which I have encountered, I can attribute none of them to other causes than defective preparatory treatment. I think I see, judging from failures to bleach at all, a strong argument in favor of the theory advanced early in the paper,—to wit, malformation of the tubuli; however, it would not be safe, in view of our lack of information and experience, to conclude that this is a fact, and thus not enter into the experiment suggested, and determine positively why we have failed in this class of discolorations. The use of chlorine gas, the agent we have employed until recently, we have concluded to be less effective than chlorine water, and shall in the future—and do now—recommend it to this body as the agent to be used in all bleaching operations.

I think that we have fallen into an error when we conclude that we can remove all that is necessary of the fatty matter by a simple application of some such agent as ammonia, borax, or bicarbonate of soda. This I believe to be impossible, and suggest the idea from the known bleaching properties of chlorine; and when we have experimented further, we will find that the elaborateness of our treatment will be confined to the primary instead of the finishing stages of the operation; at any rate, we will come down from the elaborate chlorine treatment to the apparatus mentioned earlier,—the object of which shall be the thorough cleansing of the tubuli of oleaginous matter, and when we have done this a simple application for a short time, within the canal of the tooth, of freshly-made chlorine water will be sufficient.

If you will take the trouble thoroughly to remove all fatty matters from the canal of an extracted tooth, and then immerse it in chlorine, you will find no perceptible change in its appearance even after hours of such immersion; if, however, this tooth is immersed in chlorine water, you will find that you have thoroughly and permanently restored its color.

There is no inducement for chlorine gas to penetrate to the ramification of the tubuli if the contents are dry; therefore, a very considerable force is necessary to force it through these ramifications, and the operation, so far as my experience goes, is necessarily uncertain, or at least unsatisfactory.

It is the periphery rather than the base of the tubuli that is to be treated, and we should not overlook the fact that the peripheries of the dentinal tubuli frequently extend into the enamel, making it that much more necessary that this zone should be bleached, as the nearer the labial surface the tubuli extend the surer they are to show themselves through the translucent enamel.

## Figures and Tables

**Figure f1:**